# The mixed-valent titanium phosphate, Li_2_Ti_2_(PO_4_)_3_, dilithium dititanium(III/IV) tris­(orthophosphate)

**DOI:** 10.1107/S1600536811031606

**Published:** 2011-08-17

**Authors:** Yongho Kee, Seoung-Soo Lee, Hoseop Yun

**Affiliations:** a Division of Energy Systems Research and Department of Chemistry, Ajou University, Suwon 443-749, Republic of Korea

## Abstract

The mixed-valent titanium phosphate, Li_2_Ti_2_(PO_4_)_3_, has been prepared by the reactive halide flux method. The title compound is isostructural with Li_2_Ti*M*(PO_4_)_3_ (*M* = Fe, Cr) and Li_2_FeZr(PO_4_)_3_ and has the same ^3^_∞_[Ti_2_(PO_4_)_3_]^2−^ framework as the previously reported Li_3-_ *_x_M*_2_(PO_4_)_3_ phases. The framework is built up from corner-sharing TiO_6_ octa­hedra and PO_4_ tetra­hedra, one of which has 2 symmetry. The Li^+^ ions are located on one crystallographic position and reside in the vacancies of the framework. They are surrounded by four O atoms in a distorted tetra­hedral coordination. The classical charge-balance of the title compound can be represented as Li^+^_2_(Ti^3+^/Ti^4+^)(PO_4_^3−^)_3_.

## Related literature

The synthesis and structural characterization of stoichiometric Li_2_Ti*M*(PO_4_)_3_ (*M* = Fe and Cr) and Li_2_FeZr(PO_4_)_3_ have been reported by Patoux *et al.* (2004 ▶) and Catti (2001 ▶), respectively. For related phosphates with general formula Li_3-_ *_x_M*_2_(PO_4_)_3_ (0 ≤ *x* ≤ 1), see: Wang & Hwu (1991 ▶) for Li_2.72_Ti_2_(PO_4_)_3_. For Li batteries based on Li_3-_ *_x_M*_2_(PO_4_)_3_ phases, see: Yin *et al.* (2003 ▶). For ionic conductivities of these phases, see: Sato *et al.* (2000 ▶). For ionic radii, see: Shannon (1976 ▶). For structure validation, see: Spek (2009 ▶).

## Experimental

### 

#### Crystal data

Li_2_Ti_2_(PO_4_)_3_
*M* *_r_* = 394.59
Orthorhombic, 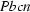

*a* = 12.0344 (5) Å
*b* = 8.5795 (5) Å
*c* = 8.6794 (4) Å
*V* = 896.14 (7) Å^3^
*Z* = 4
Mo *K*α radiation
μ = 2.39 mm^−1^
*T* = 290 K
0.22 × 0.16 × 0.14 mm

#### Data collection

Rigaku R-AXIS RAPID diffractometer
Absorption correction: multi-scan (*ABSCOR*; Higashi, 1995 ▶) *T*_min_ = 0.802, *T*_max_ = 1.000
6671 measured reflections
1004 independent reflections
974 reflections with *I* > 2σ(*I*)
*R*_int_ = 0.035

#### Refinement

*R*[*F* ^2^ > 2σ(*F* ^2^)] = 0.046
*wR*(*F* ^2^) = 0.112
*S* = 1.37
1004 reflections
87 parameters
Δρ_max_ = 0.49 e Å^−3^
Δρ_min_ = −0.74 e Å^−3^

### 

Data collection: *RAPID-AUTO* (Rigaku, 2006 ▶); cell refinement: *RAPID-AUTO*; data reduction: *RAPID-AUTO*; program(s) used to solve structure: *SHELXS97* (Sheldrick, 2008 ▶); program(s) used to refine structure: *SHELXL97* (Sheldrick, 2008 ▶); molecular graphics: locally modified version of *ORTEP* (Johnson, 1965 ▶); software used to prepare material for publication: *WinGX* (Farrugia, 1999 ▶).

## Supplementary Material

Crystal structure: contains datablock(s) global, I. DOI: 10.1107/S1600536811031606/wm2513sup1.cif

Structure factors: contains datablock(s) I. DOI: 10.1107/S1600536811031606/wm2513Isup2.hkl

Additional supplementary materials:  crystallographic information; 3D view; checkCIF report

## Figures and Tables

**Fig. 1.**
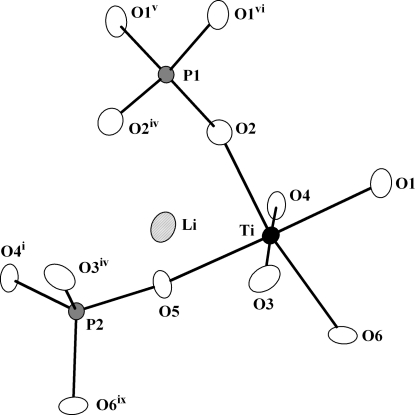
A view showing the local coordination environments of Ti and P atoms with the atom labelling scheme. Displacement ellipsoids are drawn at the 50% probability level. (Symmetry codes: (i) 0.5–x, 0.5–y, 1/2 + z; (iv) 1–x, y, 0.5–z; (v) x, 1–y, 1/2 + z; (vi) 1–x, 1–y, –z; (ix) x, –y, 1/2 + z).

**Fig. 2.**
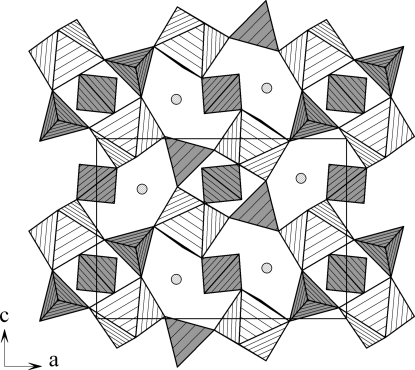
The polyhedral representation of the slab structure built up from [Ti2(PO4)3] units. Li atoms are located in the vacancies.

**Fig. 3.**
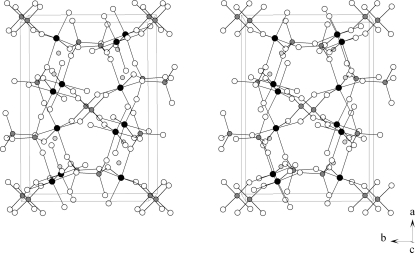
A stereoscopic view of Li2Ti2(PO4)3, viewed down the c axis.

## supplementary crystallographic information

### Comment

Lithium metal phosphates, Li_3-_*_x_**M*_2_(PO_4_)_3_, have been widely investigated as materials for secondary batteries (Yin *et al.*, 2003). It has been reported that the amount of Li can be determined in accordance with the oxidation states of metals (*M*), and Li occupancies are profoundly related to the ionic conductivity (Sato *et al.*, 2000). In attempts to control the amount of Li ions by using various metals with different oxidation states, a new member of this family with stoichiometric Li (*x*=1) has been found and we report here the synthesis and structural characterization of the mixed-valent title compound Li_2_Ti_2_(PO_4_)_3_, (I).

(I) is isostructural with mixed-metallic compounds such as Li_2_Ti*M*(PO_4_)_3_ (*M* = Fe, Cr; Patoux *et al.*, 2004) and Li_2_FeZr(PO_4_)_3_ (Catti, 2001) for which detailed investigations based on powder diffraction data have been reported. The framework of the title compound is the same as that of the previously reported Li_3-_*_x_**M*_2_(PO_4_)_3_ (0 ≤*x*≤ 1) phases such as Li_2.72_Ti_2_(PO_4_)_3_ (Wang & Hwu, 1991).

Figure 1 shows the local coordination environment of the Ti and P atoms. Two TiO_6_ octahedra are joined to three PO_4_ tetrahedra to form the [Ti_2_(PO_4_)_3_] unit. These units share a terminal oxygen atom, O4, to construct the two-dimensional slabs as shown in Figure 2. The three-dimensional framework, _∞_^3^[Ti_2_(PO_4_)_3_]^2-^ (Fig. 3) is built up from these slabs which are interconnected along the *b* axis by sharing terminal oxygen atoms O1 and O6. The Li^+^ ions reside in the vacancies and are surrounded by four O atoms in a distorted tetrahedral coordination. The Ti—O distances, ranging from 1.913 (4) to 2.045 (4) Å, are in good agreement with that calculated from their ionic radii (1.97 Å, Shannon, 1976), assuming a mixed III/IV valence.

Two crystallographically independent Li^+^ sites have been reported for Li_3-_*_x_**M*_2_(PO_4_)_3_ phases. The Li1 site is fully occupied, whereas the Li2 site is only partially occupied. The oxidation states of each metal have to be adjusted to meet the charge neutrality of these compounds. For example, the average oxidation state of Ti is +3.14 for Li_2.72_Ti_2_(PO_4_)_3_ assuming Ti to be mixed-valent (86% of Ti^III^ and 14% of Ti^IV^; Wang & Hwu, 1991)). In the title compound, the Li1 site is fully occupied, whereas the Li2 site is vacant. For charge neutrality the Ti site is occupied by 50% of Ti^III^ and 50% of Ti^IV^. Therefore, the classical charge balance of the compound can be represented by Li^+^_2_(Ti^3+^/Ti^4+^)(PO_4_^3-^)_3_.

### Experimental

The title compound, Li_2_Ti_2_(PO_4_)_3_, was prepared by the reaction of elemental Ti (CERAC 99.5%) and P (CERAC 99.5%) powders. The pure elements were mixed and loaded in a silica tube with an elemental ratio of 1:3 in the presence of LiCl (Sigma-Aldrich 99%) as a reactive flux. The mass ratio of the reactants and the flux was 1:5. The tube was kept in air for 3 days for water adsorption. It was then evacuated to 0.133 Pa, sealed, and heated gradually (60 K/h) to 1123 K in a tube furnace, where it was kept for 72 h. The tube was cooled at a rate of 5 K/h to room temperature. Air- and water-stable black block-shaped crystals were isolated after the excess flux was removed with water. Qualitative analysis of the crystals with an XRF indicated the presence of Ti and P.

### Refinement

The highest residual electron density (0.49 e Å^-3^) is 1.24 Å from the O3 site and the deepest hole (-0.74 e Å^-3^) is 2.29 Å from the O1 site. No additional symmetry, as tested by *PLATON* (Spek, 2009), was detected in this structure.

### Crystal data

**Table:**

Li_2_Ti_2_(PO_4_)_3_	*F*(000) = 764
*M**_r_* = 394.59	*D*_x_ = 2.925 Mg m^−^^3^
Orthorhombic, *P**b**c**n*	Mo *K*α radiation, λ = 0.71073 Å
Hall symbol: -P 2n 2ab	Cell parameters from 6390 reflections
*a* = 12.0344 (5) Å	θ = 3.3–27.4°
*b* = 8.5795 (5) Å	µ = 2.39 mm^−^^1^
*c* = 8.6794 (4) Å	*T* = 290 K
*V* = 896.14 (7) Å^3^	Block, black
*Z* = 4	0.22 × 0.16 × 0.14 mm

### Data collection

**Table:**

Rigaku R-AXIS RAPID diffractometer	1004 independent reflections
Radiation source: sealed tube	974 reflections with *I* > 2σ(*I*)
Graphite	*R*_int_ = 0.035
ω scans	θ_max_ = 27.4°, θ_min_ = 3.4°
Absorption correction: multi-scan (*ABSCOR*; Higashi, 1995)	*h* = −14→14
*T*_min_ = 0.802, *T*_max_ = 1.000	*k* = −11→11
6671 measured reflections	*l* = −11→11

### Refinement

**Table:**

Refinement on *F*^2^	87 parameters
Least-squares matrix: full	0 restraints
*R*[*F*^2^ > 2σ(*F*^2^)] = 0.046	*w* = 1/[σ^2^(*F*_o_^2^) + (0.*P*)^2^ + 10.7789*P*] where *P* = (*F*_o_^2^ + 2*F*_c_^2^)/3
*wR*(*F*^2^) = 0.112	(Δ/σ)_max_ < 0.001
*S* = 1.37	Δρ_max_ = 0.49 e Å^−^^3^
1004 reflections	Δρ_min_ = −0.74 e Å^−^^3^

### Special details

**Table:**

Geometry. All e.s.d.'s (except the e.s.d. in the dihedral angle between two l.s. planes) are estimated using the full covariance matrix. The cell e.s.d.'s are taken into account individually in the estimation of e.s.d.'s in distances, angles and torsion angles; correlations between e.s.d.'s in cell parameters are only used when they are defined by crystal symmetry. An approximate (isotropic) treatment of cell e.s.d.'s is used for estimating e.s.d.'s involving l.s. planes.

### Fractional atomic coordinates and isotropic or equivalent isotropic displacement parameters (Å^2^)

**Table:**

	*x*	*y*	*z*	*U*_iso_*/*U*_eq_	
Li	0.1812 (9)	0.2861 (13)	0.2191 (12)	0.020 (2)	
Ti	0.38824 (7)	0.25295 (11)	0.03788 (11)	0.0077 (2)	
P1	0.5	0.5399 (2)	0.25	0.0086 (4)	
P2	0.35246 (11)	0.10469 (15)	0.39437 (15)	0.0069 (3)	
O1	0.4200 (3)	0.3537 (5)	−0.1627 (5)	0.0186 (9)	
O2	0.4304 (4)	0.4408 (5)	0.1413 (5)	0.0219 (10)	
O3	0.5306 (4)	0.1556 (5)	0.0618 (5)	0.0217 (10)	
O4	0.2282 (3)	0.3271 (5)	0.0130 (4)	0.0152 (8)	
O5	0.3221 (3)	0.1629 (5)	0.2322 (4)	0.0137 (8)	
O6	0.3443 (3)	0.0729 (4)	−0.1040 (5)	0.0146 (8)	

### Atomic displacement parameters (Å^2^)

**Table:**

	*U*^11^	*U*^22^	*U*^33^	*U*^12^	*U*^13^	*U*^23^
Li	0.019 (5)	0.025 (5)	0.017 (5)	0.002 (4)	0.005 (4)	−0.004 (4)
Ti	0.0076 (4)	0.0079 (4)	0.0078 (4)	0.0006 (3)	0.0001 (3)	−0.0007 (4)
P1	0.0114 (9)	0.0075 (8)	0.0071 (8)	0	0.0023 (7)	0
P2	0.0060 (6)	0.0079 (6)	0.0067 (6)	0.0001 (5)	−0.0003 (5)	−0.0001 (5)
O1	0.018 (2)	0.025 (2)	0.0126 (18)	−0.0112 (17)	0.0036 (17)	−0.0017 (17)
O2	0.031 (3)	0.018 (2)	0.016 (2)	−0.0095 (18)	−0.0074 (19)	0.0006 (17)
O3	0.021 (2)	0.019 (2)	0.025 (2)	0.0074 (17)	−0.0096 (19)	−0.0075 (18)
O4	0.017 (2)	0.020 (2)	0.0087 (17)	0.0098 (16)	−0.0012 (16)	0.0001 (15)
O5	0.0107 (18)	0.021 (2)	0.0096 (17)	0.0007 (15)	0.0008 (16)	0.0035 (16)
O6	0.0131 (19)	0.0084 (18)	0.022 (2)	−0.0007 (14)	−0.0049 (17)	0.0013 (16)

### Geometric parameters (Å, °)

**Table:**

Li—O4	1.909 (11)	P1—O2^iv^	1.521 (4)
Li—O6^i^	1.979 (11)	P1—O1^v^	1.528 (4)
Li—O1^i^	1.994 (12)	P1—O1^vi^	1.528 (4)
Li—O5	2.001 (11)	P1—Li^vii^	3.048 (11)
Li—Ti^i^	2.909 (10)	P1—Li^viii^	3.048 (11)
Li—Ti	2.960 (10)	P2—O3^iv^	1.522 (4)
Li—P2	2.997 (11)	P2—O6^ix^	1.527 (4)
Li—P2^ii^	2.998 (11)	P2—O4^i^	1.531 (4)
Li—P1^iii^	3.048 (11)	P2—O5	1.537 (4)
Ti—O2	1.913 (4)	P2—Li^i^	2.998 (11)
Ti—O3	1.917 (4)	O1—P1^vi^	1.528 (4)
Ti—O1	1.981 (4)	O1—Li^ii^	1.994 (12)
Ti—O5	2.019 (4)	O3—P2^iv^	1.522 (4)
Ti—O4	2.039 (4)	O4—P2^ii^	1.531 (4)
Ti—O6	2.045 (4)	O6—P2^x^	1.527 (4)
Ti—Li^ii^	2.909 (10)	O6—Li^ii^	1.979 (11)
P1—O2	1.521 (4)		
			
O4—Li—O6^i^	131.3 (6)	O2—P1—O1^vi^	111.9 (2)
O4—Li—O1^i^	140.7 (6)	O2^iv^—P1—O1^vi^	107.1 (2)
O6^i^—Li—O1^i^	82.7 (4)	O1^v^—P1—O1^vi^	106.6 (4)
O4—Li—O5	84.2 (4)	O3^iv^—P2—O6^ix^	110.1 (2)
O6^i^—Li—O5	114.2 (5)	O3^iv^—P2—O4^i^	108.0 (2)
O1^i^—Li—O5	99.8 (5)	O6^ix^—P2—O4^i^	109.5 (2)
O2—Ti—O3	94.55 (19)	O3^iv^—P2—O5	110.8 (2)
O2—Ti—O1	89.61 (18)	O6^ix^—P2—O5	108.5 (2)
O3—Ti—O1	96.49 (19)	O4^i^—P2—O5	109.9 (2)
O2—Ti—O5	92.03 (18)	P1^vi^—O1—Ti	144.9 (3)
O3—Ti—O5	95.45 (18)	P1^vi^—O1—Li^ii^	119.3 (4)
O1—Ti—O5	167.79 (17)	Ti—O1—Li^ii^	94.1 (3)
O2—Ti—O4	92.14 (19)	P1—O2—Ti	155.2 (3)
O3—Ti—O4	172.33 (19)	P2^iv^—O3—Ti	168.1 (3)
O1—Ti—O4	87.31 (17)	P2^ii^—O4—Li	120.8 (4)
O5—Ti—O4	80.54 (16)	P2^ii^—O4—Ti	142.1 (2)
O2—Ti—O6	170.86 (18)	Li—O4—Ti	97.1 (4)
O3—Ti—O6	88.11 (17)	P2—O5—Li	115.2 (4)
O1—Ti—O6	81.39 (17)	P2—O5—Ti	142.7 (2)
O5—Ti—O6	96.43 (16)	Li—O5—Ti	94.8 (4)
O4—Ti—O6	85.86 (16)	P2^x^—O6—Li^ii^	127.8 (4)
O2—P1—O2^iv^	112.1 (3)	P2^x^—O6—Ti	138.0 (3)
O2—P1—O1^v^	107.1 (2)	Li^ii^—O6—Ti	92.6 (4)
O2^iv^—P1—O1^v^	111.9 (2)		

Symmetry codes: (i) −*x*+1/2, −*y*+1/2, *z*+1/2; (ii) −*x*+1/2, −*y*+1/2, *z*−1/2; (iii) *x*−1/2, *y*−1/2, −*z*+1/2; (iv) −*x*+1, *y*, −*z*+1/2; (v) *x*, −*y*+1, *z*+1/2; (vi) −*x*+1, −*y*+1, −*z*; (vii) *x*+1/2, *y*+1/2, −*z*+1/2; (viii) −*x*+1/2, *y*+1/2, *z*; (ix) *x*, −*y*, *z*+1/2; (x) *x*, −*y*, *z*−1/2.
